# Novel Coronavirus Disease: A delicate balancing act between health and the economy

**DOI:** 10.12669/pjms.36.COVID19-S4.2751

**Published:** 2020-05

**Authors:** Ukertor Gabriel Moti, Daniel Ter Goon

**Affiliations:** 1 Prof. Ukertor Gabriel Moti, Department of Public Administration, University of Abuja, Nigeria; 2 Prof. Daniel Ter Goon, Department of Public Health, University of Fort Hare, East London, South Africa

**Keywords:** Disruption, Economy, Health, Mitigation, Pandemic

## Abstract

The emergence of the deadly novel Coronavirus Disease (COVID-19) has resuscitated global attention on the state of health governance and well-being of citizens. Worldwide, countries are in dire dilemma of safeguarding the health of their citizens and equally salvaging economy, arising from the social distancing and lockdown strategies, which affects negatively the economic activities. This paper examines the intricate balance between health and the economy in the wake of the Covid-19 pandemic and the policy options needed to assist countries to implement strategies to protect the health of their citizens and promote economic recovery in this unprecedented global crisis situation. There is need for a coordinated public-private sector partnership in the recovery plan of each country, taking into account their contextual and country specific health system and economy, but not discarding the universal application thereof. Global, regional, national geo-political and public health collaboration is needed to save the world from COVID-19 catastrophic consequences.

## INTRODUCTION

On 31 December 2019, the Chinese government reported the novel Coronavirus (COVID-19) outbreak in Wuhan, China. When China announced citizens presenting with pneumonia symptoms, it was found that the causative agent was Severe Acute Respiratory Syndrome Coronavirus-2 (SARS-CoV-2).^1^ The World Health Organization (WHO) declared the novel Coronavirus Disease (COVID-19) as a global public health emergency on 30 January 2020;^1^ and the WHO global risk assessment of COVID-19 is very high.^3^ Currently, over 213 countries worldwide are plagued with the COVID-19 burden, with the case fatality rate of 6.87%.^4^ Worried by the risk of COVID-19, governments have adopted the Chinese lockdown model to curb its spread. Fundamentally, the lockdown measure in an outbreak of infection is to prevent people with asymptomatic infections from transmission of the disease to other persons who are not infected.^5^

The Coronavirus 2, which resulted in the COVID-19 pandemic, although dangerous, and a public health concern, is equally an independent international standard world-wide country governance assessor. It has exposed the strength or lack of institutional governance in countries worldwide: some have been found weak, non-resilient and some completely dysfunctional. There is no sector of the economy immune to the pandemic. Governments are compelled, not just to act fast and forcefully to beat the coronavirus and its economic impact, but equally to act in a manner that would guarantee effective and well-resourced public health measures to curb infection and contagion. Also there is need to implement well-targeted policies tailored towards supporting health care systems and employees. Such measures will shield the incomes of vulnerable social teams and businesses throughout the period of the virus outbreak. Understandably, immediate disruptions that may result from self-imposed shutdowns and travel bans cannot be offset by government support. Supportive economic policies, however, will revive business confidence and aid the recovery of demand as virus outbreaks ease.

Over the past decades, scholars and practitioners have analysed the impacts of epidemics and pandemics especially the socio-economic impacts and have provided insights into ways of handling pandemics.^6^-^9^ In this discourse, drawing from policy lessons from literature, we provide recommendations as governments globally battle with the delicate decision between the health and well-being of their populations and the socio-economic consequences of shutting their economies.

### Global and Regional spread of the virus

Every continent is affected by the COVID-19 pandemic. As of 27 April 2020, global confirmed cases of COVID-19 stood at 2, 858, 635, and 196, 295 deaths, across 213 countries in the world.^10^ In Africa, the confirmed cases of COVID-19 were 20, 316 and 839 deaths.^10^ While scientists search for a vaccine cure for the COVID-19 infections, non-pharmaceutical interventions can be applied─social distancing, regular handwashing, limiting unnecessary movement and social gatherings or events are crucial for the management of COVID-19.^11^ Evidence from fighting previous epidemics has proved that social distancing is effective in reducing person-to-person transmission, thereby decreasing morbidity and mortality.^12^-^14^

From a public health perspective, the upward trajectory of COVID-19 in African countries with weaker health systems is worrying. Although in principle, many African countries have expressed their readiness to manage COVID-19, which is commendable, however, practical and proactive measures regarding the availability of the rapid testing equipment, masks and protective wear for health workers is desirable. Furthermore, the respective countries’ Health Departments need to recruit more health workers to treat and manage patients in hospitals.

### Health and Economic impacts

Health is very critical to the prosperity of any society. The absence of health affects the overall well-being of an individual and limit movements. In the work place, poor health slows down individual performance levels, firm or organization productivity and ultimately income. This is what COVID-19 has caused and is causing. This disease has locked down economies and created wide-ranging socio-economic disruptions in different countries across the world. For instance, banks have lowered their repayment rates on housing loans; and there is temporary cancellation of value-added tax (VAT) receipts associated with small businesses and company taxies, amongst other `protecting and saving the economy’ measures. The unavoidable impact of all these measures is that government revenues have dwindled. Artisans and petty traders have been left without recourse whilst some small-scale businesses and even some big ones have collapsed, especially where there is total lockdown. Schools are closed resulting in months of lost education. The impact is felt across all sectors as the most effective mitigating strategies according to experts and WHO still remain hand washing, social distancing and lockdowns.^10^

From a public health perspective, health epidemics of the Middle East Respiratory Syndrome (MERS outbreak in the Asian nation) of South Korea, the Ebola virus in West Africa, and the current novel COVID-19 have international health impacts and drastic socioeconomic disequilibrium of life in the society.^15^,^16^ The unfolding of the Corona virus has aggravated an international shortage of medical equipment like medical masks, ventilators, and other Personal Protection Equipment (PPEs). Businesses are now trying to be innovative and meet these gaps in supply and demand, whilst respecting the regulations put in place.

Apart from the morbidity and mortality rates of COVID-19, the huge consequence of impaired productivity because of individual sickness, their social unit or their community are glaring.^17^ Impacts could involve psychological, academic, or skills losses on individual households. The high death rate witnessed in West Africa during the Ebola viral haemorrhagic fever outbreak triggered various impacts. Such impacts included dilated socio-economic impacts; declined growth rates; wage lost because of people’s inability to work or their contagion concerns; exaggerated economic condition and food insecurity; loss of education and lost jobs.^18^ A similar trend is emerging with COVID-19. The global infection rate is high and increasing exponentially, and only the early preventative measures imposed by governments on populations could possibly slow down the death rates. However, some of the preventative measures directly or indirectly affect the economy negatively.

### Policy changes to scale back uncertainty and improve growth prospects

Given the already weak economy of some countries and the resultant draw back risks precipitated by the COVID-19 pandemic, the near-term challenges would warrant reinforcing some policy actions to contain the untold challenges of COVID-19. There is a need to strengthen health care systems, boost investor confidence and demand, and limit adverse economic effects. There is need for multilateral policy dialogue to curtail and foster agreements on acceptable containment. Policy measures are needed to cushion the economic impact of the coronavirus regarding inflation of essential commodities and services.

As countries grapple with mitigation and containment, the socio-economic impact is affecting the containment measures which when relaxed, may have dire health consequences. This has called for a delicate balance between health and economic considerations. There are no easy choices. Each country will have to carefully navigate this dilemma within its context, but based on what the prevailing figures and available scientific evidence from experts reveals.

Broadly, however, governments need two policy instruments to deal with the pandemic: A mitigating policy and a Post Covid-19 recovery and rejuvenation policy. The mitigation policy will be a Health policy-a Proactive Health Policy framework and Management approach. This will involve containment measures; protection of health providers (provision of Personal Protection Equipment (PPEs) and incentives); Testing centres and kits; Isolation centres with relevant facilities etcetera), in addition to aggressive education and health literacy.

Health education and literacy is important to mitigate the impact of COVID-19 on crises-affected populations. Some countries may have to create massive information and surveillance: people would need to provide a transparent image of travel history, health records, movement and different relevant data so as to assist identification of (potential) infected persons. Therefore, governments and agencies can implement evidence-based approaches recommended by the World Health Organisation (WHO) for combating Covid-19. Hand washing hygiene could be an extremely effective public health intervention, amidst other preventative measures such as regular use of facial masks, safe coughing and social distancing.^19^

The post Covid-19 recovery and rejuvenation policy or economic policy should include palliatives to encourage citizens to endure lockdowns and observe social distancing. To cushion adverse effects of the Coronavirus on vulnerable social teams, short-time operating schemes, could be implemented to boost the pliability of operating hours. Such schemes would protect jobs and pay, but they may not be pliable enough to defend temporary or migrant staff from lay-offs.^20^

Governments can adapt several economic palliative measures to mitigate the impact of Covid-19 on households. These could include providing money transfers or state insurance, especially for staff placed on unpaid leave and guarantees for lower medical costs for all. Additionally, governments can reduce policy interest rates and possibly, provide economic stimulus packages to boost business confidence. Such measures would assist with the recovery of demand, once the spread of the virus eases and travel restrictions are lifted. However, such measures could be less effective in coping with the immediate supply-side disruptions that result from imposed shutdowns and travel restrictions.

## CONCLUSION

Discernably, this paper recommends coordinated public-private sector partnership recovery policy agenda with all stakeholders in health and business anchored on scientific evidence regarding balancing health and the economy that aligns to an informed systems approach and cost-sharing strategies in re-opening of businesses, without possible resurgence of the virus. This approach entails phased opening of the economy and educational institutions where the effect of the pandemic is wide spread. Importantly, there must be synergy between the Health policy and the Economic and Post COVID-19 recovery policy in order not to tip the delicate balance between health and economy. While some of these policy strategies and approaches may be contextual and country specific, they are nonetheless universal. The time for international geo-political and public health collaboration and solidarity is now and the world must save no resources in protecting regional, national and international populations and economies. Now is the time to spend to save!

### Authors’ Contribution

**UGM** conceived and wrote the first draft

**DTG** read and edited the manuscript and is responsible and accountable of the study.

All authors contributed to the writing of the manuscript, read and approved the final version of the manuscript.
